# Editorial: Epilepsy in veterinary science

**DOI:** 10.3389/fvets.2023.1200311

**Published:** 2023-05-17

**Authors:** Andrea Fischer, Holger Andreas Volk

**Affiliations:** ^1^Centre for Clinical Veterinary Medicine, Ludwig-Maximilians-Universität München, Munich, Germany; ^2^Klinik für Kleintiere, Stiftung Tierärztliche Hochschule Hannover, Hannover, Germany

**Keywords:** canine idiopathic epilepsy, functional neuroimaging, neurostimulation, epilepsy surgery, feline epilepsy, animal model, pharmacoresistance, treatment

Epilepsy is a unique manifestation of abnormal network excitability of the brain of humans and animals, which remains poorly understood, and, therefore, challenging to treat, presenting with an ongoing and frequently life-long predisposition for epileptic seizures. Epilepsy occurs in dogs with a similar population prevalence to humans. Broad etiologic classes are largely shared between the two species. Etiologies range from a few monogenic inherited genetic epilepsies with onset in childhood and juveniles, the presence of genetic risk variants and risk haplotypes in association with epilepsies without identifiable lesions on brain MRI (unknown etiology), and structural epilepsies, e.g., from brain tumors, post-traumatic epilepsy, and malformations of cortical development (1, 2).

Autoimmune encephalitis and autoimmune-associated epilepsy form a new distinct etiologic group in humans offering new treatment approaches. In humans, autoimmune encephalitis is frequently associated with neural antibodies, presents with new onset of epileptic seizures, psychiatric and behavioral symptoms, and subtle inflammatory CSF and MRI changes, and may cause new-onset refractory status epilepticus (NORSE) (3). Huenerfauth et al. contribute evidence for the involvement of GABA-A receptor antibodies in new-onset refractory status epilepticus (NORSE) in dogs and parallels to humans (4). Five different neural antibodies have been described in dogs up to now including this case report (NMDAR1, GFAP, GAD65, IgLON5, GABA-A receptor antibodies). Yet their clinical impact is not always as clear as for LGI1 antibodies in cats with limbic encephalitis (5, 6), and more research is necessary to explore disease associations. This is especially important considering the frequent occurrence and the efforts and challenges of the treatment of status epilepticus in dogs (7), and the potential for treatment with anti-inflammatory or immunosuppressive drugs.

There is an urgent need to develop and evaluate better therapies for canine epilepsy, considering the high proportion of dogs with idiopathic epilepsy (IE) and to drug-resistant epilepsy (8–10). The need to step forward in canine epilepsy is reflected by the manuscripts submitted to the Research Topic “*Epilepsy in veterinary sciences*.” Twenty-two manuscripts were published on this Research Topic, 19 focused on canine epilepsy, and 18 on treatment aspects of epilepsy. Overall, the type of manuscripts ranged widely from single case reports to observational and explorative cohort studies (phenotype, biomarkers, physiologic responses, seizure detection), to reviews and prospectively controlled treatment studies. All contributed significantly to knowledge in the field. Most manuscripts of the Research Topic collection, “*Epilepsy in veterinary science*” explored treatment strategies and potential biomarkers for treating epilepsy in dogs. These manuscripts highlight the relevance of difficult-to-treat epilepsy in dogs and the need to develop novel treatment strategies for client-owned dogs with IE.

The genetic background of many dog breeds appears to predispose them to epilepsy with a severe clinical course. The appearance of tonic-clonic seizure in clusters appears as a point-of-no-return across many breeds (9). Breed-specific epilepsies in Greater Swiss Mountain dogs and Border Collies were the focus of three manuscripts of this article collection. All of them considered treatment response, survival, and quality of life an important aspect of the epilepsy description. First, Ostermann et al. confirmed a severe manifestation of IE with frequent tonic-clonic seizures in a large cohort of Greater Swiss Mountain dogs. In addition, they proved the frequent occurrence of cluster seizures and status epilepticus, significantly impacting survival time. Their finding aligned with previous studies on the association of cluster seizures with drug refractoriness (9). Santifort et al. explored a cohort of 116 Border Collies with IE and confirmed the previously found association between a younger age of onset and a more severe manifestation of epilepsy. This finding raises the question, of whether the age of onset should be more closely explored in dogs as a characteristic feature of epilepsy syndromes (11). It is a concern that a significant percentage of owners perceived the quality of life of their dogs as heavily impacted. Finally, Mauri et al. explored the role of 3T single voxel magnetic resonance spectroscopy to track changes in brain metabolites in these two breed-specific epilepsies (Border Collies, Greater Swiss Mountain dog) in a prospective cohort study. N-acetyl aspartate (NAA) was used as a marker for neuronal degeneration and glutamate-glutamine for neuronal excitability. Their results showed decreased glutamate-glutamine in thalamic regions in treated dogs compared to untreated dogs and controls. There is a need for more functional neuroimaging studies in veterinary epilepsy (see also review provided by Szabo and Salinas in this Research Topic).

Large breed dogs show IE more frequently than small breed dogs offering the chance to apply neurostimulation devices and strategies designed for use in humans. Previously, Charalambous et al. showed the potential efficacy of repetitive transcranial magnetic brain stimulation (rTMS) in drug-resistant dogs with IE (12). Yet more data are necessary for a targeted approach e. g., to define the targeted brain areas and changes in the canine brain networks in space and time. Interestingly, 2-deoxy-2-[^18^F] fluoro-D-glucose positron emission tomography (18F-FDG-PET) could demonstrate differential effects of precisely targeted high-frequency rTMS on glucose metabolism in the pre-frontal cortex and hippocampus of healthy Beagle dogs (13). The upcoming potential of neurostimulation for the non-pharmacologic treatment of seizures in dogs is the focus of three manuscripts within the Research Topic “*Epilepsy in veterinary science*”: A review by Nowakowska et al. summarizes current knowledge on available neurostimulation methods for treatment of drug-resistant epilepsy in dogs, and highlights repetitive transcranial magnetic and transcutaneous vagal nerve stimulation. Castillo et al. documented. changes in electroencephalography (EEG) power spectra, increased heart rate variability, and decreased heart rate after transcutaneous vagal nerve stimulation in healthy Beagle dogs. EEG was recorded with subdermal wire electrodes for1 h before and 1 h after transcutaneous vagal stimulation for 120 s with a device marketed for dogs. Further exploration of this approach in controlled studies is warranted. Notably, Hirashima, Saito, Igarashi et al. reported on the successful use and long-term follow-up of an implanted vagal nerve stimulator in a dog with drug-resistant IE. There was a significant (> 50%) and long-lasting decrease in tonic-clonic seizures but less impact on focal seizures. No side effects other than coughing occurred. The owner was also provided with an external magnet that could induce a vagal pulse that could prevent focal seizures' evolution to tonic-clonic seizures.

A series of three manuscripts provide insights into metabolic alterations of current treatment strategies with phenobarbital or medium-chain triglycerides (MCT) in dogs with IE: Watanagura et al. investigated alterations in the microbiota-gut-brain axis and behavioral changes that occurred in phenobarbital-treated dogs in a prospective cohort study. Analysis of fatty acids with gas chromatography mass spectrometry (GC-MS) indicated an association between the short-chain fatty acid butyrate and drug responsiveness in new-onset epilepsy (Watanagura et al.). This opens exciting research perspectives on fecal short-chain acids as potential biomarkers of drug responsiveness). Berk et al. explored the metabolite and neurotransmitter profiles in a prospective well-controlled study with a cross-over design in dogs with IE and documented changes in amino acids, fatty acids, and neurotransmitters associated with dietary MCT oil supplements. Furthermore, the ß-hydroxybutyrate-triacylglyceride ratio showed an overall negative correlation with seizure frequency. In support of this study, Schmidt et al. further explored altered urinary neurotransmitter profiles in dogs with IE.

Lastly, the Research Topic “*Epilepsy in veterinary science*” includes also studies, which provide data on the potential efficacy of new treatment strategies in dogs with drug-resistant IE. Polidoro et al. investigated the pharmacokinetics of an intranasal, rectal and oral administration of a cannabidiol (CBD) formulation in six healthy Beagle dogs. Fischer et al. explored the efficacy of a cyclooxygenase-2 inhibitor (firocoxib) as an add-on treatment in dogs with phenobarbital-resistant epilepsy in a translational pilot study. The research hypothesis originated from rodent data on seizure-induced induction of p-glycoprotein at the blood-brain barrier. Considering, that only the two dogs with the highest seizure frequency were partial responders with a >50% decrease in seizures (18.2% per protocol, 11.8% intent-to-treat) argued against the overall efficacy of the add-on treatment (Fischer et al.). Subsequently, Kriechbaumer et al. introduced a time-to-event study design for dogs with drug-resistant epilepsy. Thereby dogs participated in the study only until their pre-defined clinical trial endpoint (n-th seizure). This protocol aimed to reduce the study duration of non-responders. The efficacy evaluation follows International Veterinary Epilepsy Task Force (IVETF) suggestions (14). The International League Against Epilepsy regulatory task force and pediatric commission proposes a similar approach where children participate until the same number of seizures occurs as in a predefined baseline period (15–17). Both canine studies demonstrate a lack of relevant placebo responses in drug-resistant dogs, contrary to previous findings (18).

Other manuscripts explored current medical treatment strategies in dogs with reactive (acute symptomatic) seizures or IE: efficacy of levetiracetam as first-line treatment of presumed reactive seizures from exogenous poisoning by Stabile et al., variables with a potential influence on treatment with bromides in 220 dogs from the Netherlands by Lichtenauer et al.; aggressive behavior as an adverse event of zonisamide by Kanazono et al., the approach of primary care UK veterinarians toward the management of canine IE by Griffin et al..

The call for papers for the Research Topic “*Epilepsy in veterinary science*” addressed originally also other animal species than dogs. Spontaneous epilepsy has been reported in many other animal species, and animal models are widely used as research tools in epilepsy. Yet contributions from different species were limited to three manuscripts on cats and baboons. Hasegawa, Asada, Hamamoto et al. and Hasegawa, Asada, Mizuno et al. report on their efforts to cure drug-resistant epilepsy in two cats with epilepsy surgery. Their approaches were individualized to the cats' epilepsy type. Hippocampectomy was chosen in one cat, and corpus callosotomy in the other cat (Hasegawa, Asada, Hamamoto et al. and Hasegawa, Asada, Mizuno et al.). The manuscripts highlight the challenges of identifying the epileptogenic zone in chronic drug-resistant epilepsy, even if structural lesions are visible on MRI. Szabo and Salinas provide a review of neuroimaging in baboons. Baboons with spontaneous epileptic seizures are a large animal model for idiopathic generalized epilepsies in humans and photosensitivity. A similar genetically defined generalized idiopathic epilepsy has been described in Rhodesian Ridgebacks (19). The review provides insights into the perspectives of structural and functional neuroimaging in a large animal species. While structural imaging is usually standard in individual animals with idiopathic generalized epilepsies, statistical approaches can identify gray matter volume/concentration changes. Functional neuroimaging can map epileptic networks, altered functional connectivity of physiological networks, photoepileptic responses, and the effects of anti-seizure therapies (Szabo and Salinas).

Research for better treatment of epilepsy in dogs and cats is driven by the strong human-animal bond, the efforts, and the psychological and emotional stress owners face when caring for an animal with epilepsy beyond the financial issues involved. The power of this human-animal bond can drive efforts to better our treatment armory of epilepsy. Novel seizure detection devices will provide objective data on tonic-clonic seizure counts, which can supplement the reports of the owners in treatment studies in veterinary epilepsy. The data provided by Hirashima, Saito, Kuriyama et al. show the potential of this approach. Like in human epilepsy, in addition to drug treatment, the new era of epilepsy treatment in pets also includes dietary, neurostimulation, and surgical options. The hope is that the convergence of human and veterinary research needs will enhance awareness and funding of epilepsy research in dogs and cats (1).

## Author contributions

AF drafted the work. HV provided review and advice. Both authors are responsible for the content. Both authors contributed to the article and approved the submitted version.
